# Nox2 impairs VEGF-A-induced angiogenesis in placenta *via* mitochondrial ROS-STAT3 pathway

**DOI:** 10.1016/j.redox.2021.102051

**Published:** 2021-06-18

**Authors:** Chengjun Hu, Zifang Wu, Zihao Huang, Xiangyu Hao, Shuqi Wang, Jinping Deng, Yulong Yin, Chengquan Tan

**Affiliations:** a Guangdong Laboratory for Lingnan Modern Agriculture, Guangdong Provincial Key Laboratory of Animal Nutrition Control, National Engineering Research Center for Breeding Swine Industry, Institute of Subtropical Animal Nutrition and Feed, College of Animal Science, South China Agricultural University, Guangzhou, Guangdong, 510642, China; b Tropical Crops Genetic Resources Institute, Chinese Academy of Tropical Agricultural Sciences, Haikou, 571101, China; c National Engineering Laboratory for Pollution Control and Waste Utilization in Livestock and Poultry Production, Institute of Subtropical Agriculture, Chinese Academy of Sciences, Changsha, Hunan, 410125, China

**Keywords:** Angiogenesis, IUGR, Nox2, Placenta, STAT3, VEGF-A

## Abstract

Aberrant placental angiogenesis is associated with fetal intrauterine growth restriction (IUGR), but the mechanism underlying abnormal placental angiogenesis remains largely unknown. Here, lower vessel density and higher expression of NADPH oxidases 2 (Nox2) were observed in the placentae for low birth weight (LBW) fetuses versus normal birth weight (NBW) fetuses, with a negative correlation between Nox2 and placental vessel density. Moreover, it was revealed for the first time that Nox2 deficiency facilitates angiogenesis *in vitro* and *in vivo*, and vascular endothelial growth factor-A (VEGF-A) has an essential role in Nox2-controlled inhibition of angiogenesis in porcine vascular endothelial cells (PVECs). Mechanistically, Nox2 inhibited phospho-signal transducer and activator of transcription 3 (p-STAT3) in the nucleus by inducing the production of mitochondrial reactive oxygen species (ROS). Dual-luciferase assay confirmed that knockdown of Nox2 reduces the expression of VEGF-A in an STAT3 dependent manner. Our results indicate that Nox2 is a potential target for therapy by increasing VEGF-A expression to promote angiogenesis and serves as a prognostic indicator for fetus with IUGR.

## 1 Introduction

Intrauterine growth restriction (IUGR) is a complication of pregnancy leading to low birth weight (LBW). LBW fetuses had a lower growth rate and an increased incidence of diseases, such as obesity, diabetes, insulin resistance, hypertension, and cardiovascular disease later in life [1]. Although many factors, such as maternal nutrition, flow of blood, and placental dysfunction have been reported associated with LBW [2,3], the mechanism of LBW is still unclear. Therefore, it is important to explore the mechanism to obtain better prevention outcomes in fetuses with LBW.

The formation of placental blood vessels is important for fetal growth. Previous studies have revealed that impaired angiogenesis in LBW placentae [4,5], indicating that poor placental blood vessel development weakens the exchange of oxygen/nutrients and blood flow, thus reducing fetal growth [6]. Vascular endothelial growth factor-A (VEGF-A), an important angiogenic factor [7], binding to the VEGFR receptor-2, promotes the activation of tyrosine kinase enzyme, which can activate several intracellular signaling pathways, thus producing several biological effects, including proliferation, migration, and angiogenesis [8]. Previous studies confirmed the significant downregulation in the VEGF-A protein level in LBW placenta [9], indicating that decreased VEGF-A might contribute to abnormal angiogenesis in IUGR placenta. However, the mechanism needs further investigation.

High oxidative stress was shown to impair cell function and angiogenesis [10,11]. NADPH oxidase 2 (Nox2), as part of a NADPH oxidase complex, is a major source of reactive oxygen species (ROS) in endothelial cells [12], and it also plays a vital role in angiogenesis. For instance, Nox2-derived ROS contribute to hypercholesterolemia-induced inhibition of neovascularization [13], and Nox2 deficiency protects against age-dependent impairment of neovascularization in muscle [14]. However, the role of Nox2 in modulating placental angiogenesis is currently unknown. The purpose of the present study was to use the model of LBW and normal body weight (NBW) piglet placentae to investigate the role and the underlying mechanism of Nox2 in placental angiogenesis. We found that higher expression of Nox2 was observed in the LBW than in the NBW placentae. Moreover, knockdown of Nox2 was found to significantly increase angiogenesis both *in vitro* and *in vivo*. Furthermore, the role of Nox2 in angiogenesis was shown to depend on the STAT3/VEGF-A signaling pathway in porcine vascular endothelial cells (PVECs). Our findings reveal that Nox2 plays a critical role in placental angiogenesis and can serve as a prognostic indicator for IUGR.

## 2 Materials and methods

### 2.1 Placental samples

**Fig. 1:**
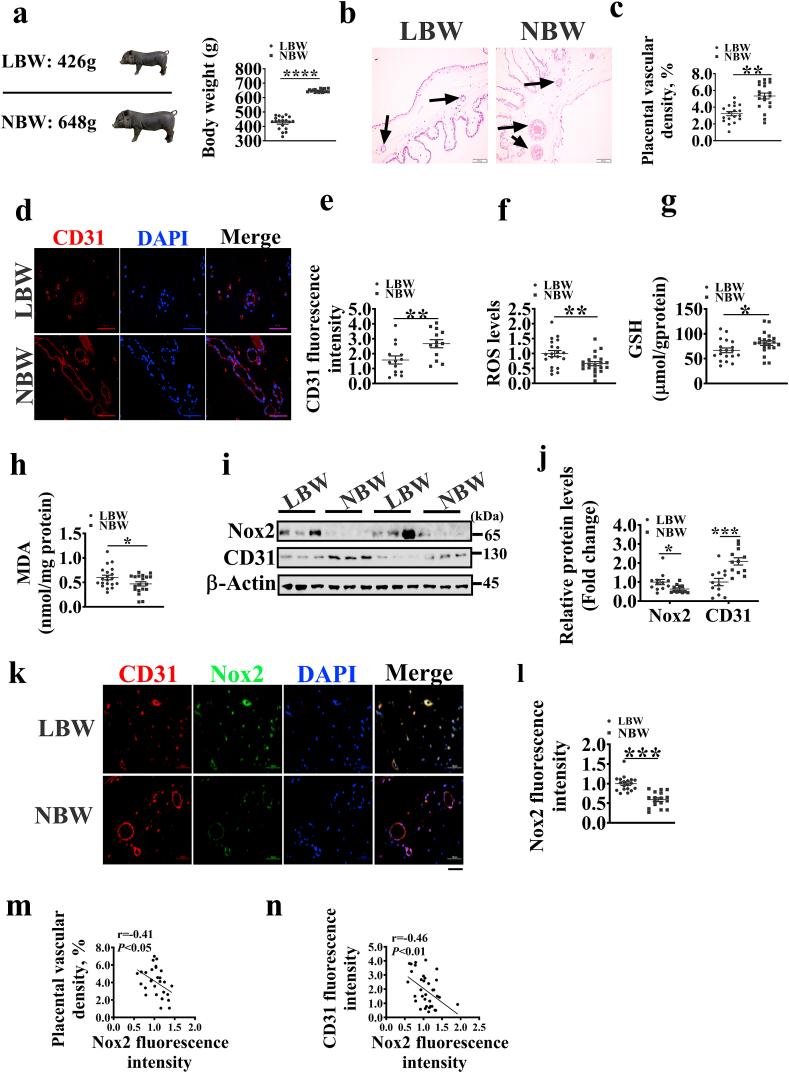
Changes of vessel density and Nox2 in placentae for low (LBW) and normal (NBW) birth weight piglets. a. The birth weight of piglets in LBW and NBW groups (n = 19). b. H&E staining to show vessel structures in placentae; the black arrows indicate the placental vessels. Scale bar, 200 μm. c. Histograms indicating placental vascular density in each group (n = 19). d. CD31 staining to show the distribution of vessel density in placentae. Scale bar, 50 μm. (e) Histograms indicating CD31 fluorescence intensity in each group (n = 14). f-h. The ROS, MDA, and GSH levels in placentae (n = 18). i. Protein levels of Nox2 and CD31 in placentae. j. Histograms indicating Nox2 and CD31 protein levels in each group (n = 12). k. Immunofluorescence images showing that Nox2 is highly expressed in blood vessels with low CD31 immunofluorescence density. Scale bar, 50 μm. l. Histograms indicating Nox2 fluorescence intensity in each group. m, n. Correlation between placental vascular density or CD31 fluorescence intensity and Nox2 fluorescence intensity. Data represent the mean ± SEM, *P < 0.05, **P < 0.01, ***P < 0.001.

In this study, a total of 339 neonatal piglets were obtained, with an average birth weight of 642.2 ± 9.6 g (means ± standard error). Next, piglets with birth weight less than 500g were assigned to the low birth weight group (average birth weight = 426 g, LBW group), and those between 600g and 700g were assigned to the normal birth weight group (average birth weight = 648 g, NBW group) (Fig. 1a). The placentae for LBW and NBW group piglets were weighed and collected, and ~ 2 g of each placenta was immediately snap-frozen in liquid nitrogen or immediately fixed in 4% paraformaldehyde. The experimental design and procedure adopted in this study were reviewed and approved by the Animal Care and Use Committee of South China Agricultural University.

### 2.2 Hematoxylin-eosin (H&E) and immunofluorescence staining

Placental tissues fixed in 4% paraformaldehyde were paraffin-embedded and sectioned at 5 μm thickness, followed by staining with hematoxylin-eosin (H&E). The area occupied by placental tissues was traced and the placental vessels in these areas were also traced using a microscope (Olympus CX41, Japan). Next, the placental vascular areas were quantified *via* image analysis and evaluated for the relative number of placental vessels per unit tissue area. The remaining sections were used for platelet endothelial cell adhesion molecule-1 (CD31) and Nox2 immunofluorescence staining as described in our previous work [15]. Three photographs were selected randomly for each slide using a fluorescent microscope (Nikon Eclipse C1, Tokyo, Japan). The fluorescence intensities of CD31 and Nox2 were quantified using Image J software (National Institutes of Health, Bethesda, MD).

For staining PVECs, the cells were plated in slide with 2 mL medium containing 10% fetal bovine serum. After staining for 24 h, the cells were fixed in 4% paraformaldehyde for 10 min, followed by incubation at room temperature overnight in a wet box with the following primary antibodies: Goat anti‐rabbit p-STAT3 (Abcam, USA), goat anti‐mouse VEGF-A antibody (Novus, USA), goat anti‐rabbit Nox2 (Proteintech, USA). Nuclei were stained with 4′,6-diamidino-2-phenylindole (DAPI).

### 2.3 Cell culture

PVECs were purchased from the Cell Bank of the Chinese Academy of Sciences (Shanghai, China), and cultured at 37 °C with 5% CO_2_ in 1640 medium with 10% fetal bovine serum, 100 U/mL penicillin, and 100 μg/mL streptomycin.

### 2.4 Small interfering RNA (siRNA) and adenovirus vector transfection

PVECs were grown to 50% confluence in 6-well plates and transfected with Lipofectamine 2000 (Invitrogen, Carlsbad, CA, USA) as instructed by the manufacturer. Briefly, PVECs were seeded in 6-well plates at 2 × 10^4^ cells/well and incubated for 24 h, followed by transfection with 20 nM si Ctrl and si Nox2 for 6 h. After transfection, the medium was replaced with RIPM1640 containing 10% FBS and then incubated for 48 h. Finally, Western blotting was used to assess the expression of Nox2. The sequences were 5′-CGCUGUGUCUCAUAUUAAUTT-3′ and 5′-AUUAAUAUGAGACACAGCGTT-3′ for Nox2 siRNA; 5′-CCACUGAGGAGUUCAACAUTT-3′ and 5′-AUGUUGAACUCCUCAGUGGTT-3′ for VEGF-A siRNA; 5′-UUCUCCGAACGUGUCACGUTT-3′ and 5′-ACGUGACACGUUCGGAGAATT-3′ for control siRNA. The siRNA was purchased from GenePharma Co., Ltd (Shanghai, China).

For adenovirus transfection, PVECs were plated in 6-well plates, and at 70–80% confluence, the cells were infected with adenovirus at a multiplicity of infection (MOI) of 50. After infection for 6 h, the medium was replaced with RIPM1640 containing 10% FBS and then incubated for 48 h. Finally, Western blotting was used to assess the protein expression level of Nox2. The Ad-Nox2 adenovirus was generated from GenePharma Co., Ltd. (Shanghai, China).

### 2.5 Oxidative stress parameters

The reactive oxygen species (ROS) level in placentae and cells was measured using 2′, 7′-dichlorofluorescein diacetates (DCFH-DA) according to the manufacturer's protocol (Nanjing Jiancheng Bioengineering Institute, Nanjing, China) as previously described [16]. Briefly, PVECs were seeded in 96 well plates after si Ctrl and si Nox2 transfection. At 60–70% confluency, cells were starved with serum free media for 4 h, followed by adding RIPM1640 with 10% FBS and 20 μM DCF-DA to the cells and incubation for 2.5 h at 37 °C.

Mitochondrial ROS level was measured using MitoSOX Red (Invitrogen, Carlsbad, USA) assay. PVECs were seeded in 96 well plates with a density of 5 × 10 ^4^/mL and incubated for 24 h, followed by transfection with 20 nM si Ctrl and si Nox2 for 6 h. After transfection, cells were incubated with 5 mmol/L MitoSOX Red for 10 min at 37 °C. After two washes with PBS, the images of the cells were captured with an Olympus inverted microscope and analyzed using the Image J software.

Malondialdehyde (MDA) was quantified using the thiobarbituric acid (TBA) method according to the manufacturer's protocol (Nanjing Jiancheng Bioengineering Institute, Nanjing, China). Glutathione (GSH) in placenta was determined using the commercial kits (Nanjing Jiancheng Bioengineering Institute, Nanjing, China). Placental total protein concentrations were measured according to the instructions of the bicinchoninic acid (BCA) protein assay kit (Beyotime, Beijing, China). The placental MDA and GSH levels were normalized to placental total protein.

### 2.6 Tube formation assay

A total of 4 × 10^4^ PVECs were seeded in 96-well plates pre-coated with 50 μL Matrigel (BD company, USA) and incubated with serum-free medium or conditioned cell culture medium at 37 °C for 6 h. Then the images were captured using an Olympus inverted microscope (40 × ) and the results of tube formation were analyzed using Image J software.

### 2.7 Transwell assay

PVECs were collected and suspended in serum-free medium. Then, the cells (4 × 10^4^) were seeded into the upper chamber, followed by adding 600 μL of serum-free medium or conditioned cell culture medium to the lower chamber. Next, the PVECs were incubated for 24 h and then stained with crystal violet solution. Images were captured with an Olympus inverted microscope and analyzed using the Image J software.

### 2.8 Wound healing assay

PVECs were seeded into 6-well plates. After siRNA transfection or inhibitor treatment, the PVECs were wounded with a 10 μL pipette tip and maintained for 24 h in basal medium or conditioned cell culture medium. The wounded areas at 0 h and 24 h were captured using an Olympus inverted microscope and quantified using the Image J software.

### 2.9 ELISA assay

The culture supernatants were collected for VEGF-A quantitation. The VEGF-A level in the supernatants was measured through ELISA (CSB-E12053p, Cusabio, Wuhan, China, https://www.cusabio.com/) according to the manufacturer^'^s protocol.

### 2.10 Quantitative real-time PCR

Total RNA was isolated from the PVECs using the TRIzol reagent (Invitrogen, Carlsbad, USA). cDNA synthesis was performed using the PrimeScript RT reagent kit (Takara, Dalian, China). The primers used are shown in Supplemental Table S1. The PCR reaction was performed using ABI QuantStudioTM 6 Flex system (Applied Biosystems, Carlsbad, CA) and SYBR Green Reagent kit (GenStar, Beijing, China). The relative gene expression was expressed as a ratio of the target gene to the control gene, with 18S rRNA used as a control gene.

### 2.11 Nuclear and cytoplasmic protein extraction

The nuclear and cytoplasmic proteins were extracted according to the procedure of the kit (Sangon Biotech, Shanghai, China).

### 2.12 Western blotting

Placentae and cells were lysed by RIPA buffer (Beyotime, Beijing, China) containing protease and phosphatase inhibitors. The protein concentrations were determined using a BCA Protein Assay kit (Beyotime, Beijing, China) according to the manufacturer's guide, and then separated by SDS-PAGE and blotted onto PVDF membranes. Next, the blots were incubated at 4 °C overnight with the following primary antibodies: VEGF-A (Proteintech, USA), VEGFR2 (Absin Bioscience, Inc., Shanghai, China), phospho-VEGF Receptor 2 (Tyr1175) polyclonal antibody (CST, USA), Nox2 (Proteintech, USA), β-Actin (CST, USA), p-STAT3 (Abcam, USA), STAT3 (Abcam, USA), CD31 (Abcam, USA), and Histone H3 (Proteintech, USA). The density of bands was quantified using Image J software (National Institutes of Health, Bethesda, MD) and then normalized to β-Actin.

### 2.13 Protein stability assay

A cycloheximide (CHX) -based assay was used to analyze the protein turnover of p-STAT3 in PVECs. Briefly,12 μg/mL CHX was added to cell culture medium and cells were harvested at the indicated time points. Next, the cells were lysed by RIPA buffer (Beyotime, Beijing, China) containing phosphatase inhibitors, followed by Western blotting the cell lysates with anti‐p-STAT3 and anti‐β‐actin as indicated. Finally, the p-STAT3 protein levels relative to β‐Actin were quantified using the Image J software.

### 2.14 Co‐immunoprecipitation (co‐IP) assay

PVECs were harvested and washed three times by PBS, followed by lysing the cells with RIPA buffer (Beyotime, Beijing, China), obtaining the cell lysates by centrifugation, and incubation for 2 h at 4 °C with 2 μg of indicated antibody and protein A + G Magnetic beads (Beyotime, Beijing, China). After three washes with buffer, the immunocomplexes were separated by SDS-PAGE.

### 2.15 Luciferase reporter assay

The pig STAT3-binding motif VEGF-A promoter was cloned into the pGL3 reporter plasmid (Supplemental Fig. S4d). Mutations were introduced into the sequences of the VEGF-A promoter to generate VEGF-A mutation reporters. The Renilla luciferase reporter plasmid (pRL-TK) was co-transfected as an internal control plasmid. After 48-h transfection, the cells were lysed and assayed. The luciferase activity of VEGF-A promoter was measured using the Dual-Luciferase Reporter kit (Promega, Madison, WI, USA).

### 2.16 Mitochondrial ROS level and membrane potential determination

The mitochondrial ROS level in PVECs was determined using MitoSOX Red (Invitrogen, USA). Briefly, PVECs were plated on 24-well plates and incubated with 5 μM MitoSOX Red at 37 °C for 10 min. Immunofluorescence images were captured using an Olympus inverted microscope.

The mitochondrial membrane potential was determined using the mitochondrial membrane potential assay kit (Beyotime, Beijing, China) according to the manufacturer's instructions.

### 2.17 Intracellular adenosine triphosphate (ATP) and mitochondrial DNA (mtDNA) levels

Intracellular ATP levels were measured using an ATP assay kit (Beyotime, Shanghai, China) according to the manufacturer's instructions.

Total genomic DNA of PVECs was isolated from the placenta using the QIAamp DNA Mini Kit (Qiagen, USA). mtDNA levels were determined using primers for mitochondrial cytochrome *b* (Cytb), and normalized to genomic DNA by amplification of the 18S rRNA [15].

### 2.18 In vivo matrigel plug assay

Male BALB/c nude mice were purchased from Guangdong Medical Laboratory Animal Center (Foshan, Guangdong, China) and allowed to acclimate for 1 week before use. Then, the BALB/c nude mice (6 weeks of age) were given subcutaneous injections of 400 μL of Matrigel (BD Biosciences, USA) containing 100 μL CM from siCtrl or siNox2 cells treated with or without 5 μM Stattic. 15 days later, the Matrigel plugs were dissected out and used for hemoglobin measurement or CD31 staining. All experiments were approved by the Care and Use of Laboratory Animal Center and Animal Ethics Committee of South China Agricultural University.

### 2.19 Hemoglobin assay

The hemoglobin concentration in the Matrigel plugs was measured using a commercial kit (Bestbio, Shanghai, China). Briefly, the plugs were homogenized in 400 μL of RIPA lysis buffer and centrifuged at 3000 rpm for 15 min at 4 °C to collect the supernatant. Finally, the supernatant was used for hemoglobin determination according to the manufacturer's instructions.

### 2.20 Statistical analysis

The differences between the two groups were analyzed by the Student's *t*-test using SPSS 20.0 (SPPS Inc., Chicago, IL) software. Experiments were repeated three to five times. Data were presented as mean ± SEM. The Pearson correlation analysis was performed to determine the correlation among the groups. *P* < 0.05 was considered as statistically significant.

## 3 Results

### 3.1 Nox2 is highly expressed in LBW placentae and negatively associated with placental vessel density

Placental vessels play an important role in fetal growth and development. The placentae for LBW fetuses showed a lower weight than those for NBW fetuses (Supplemental Fig. S1a). To investigate the placental angiogenesis in LBW and NBW, we analyzed the placental vessel density using H&E (Fig. 1b) and immunofluorescence staining (Fig. 1d). The results showed that the vessel density was lower in the LBW group than in the NBW group (Fig. 1c). CD31, a marker of endothelial cells in blood vessels [17], was highly expressed in the NBW group (Fig. 1e). Here, we also observed that the LBW group was higher than NBW group in the ROS and MDA levels (Fig. 1f, h), but lower than NBW group in the GSH level (Fig. 1g). Nox2 and Nox4 are the major sources of ROS in endothelial cells [18]. Our previous study has shown that Nox2 is the main isoform of NADPH oxidase in the placenta [15]. To examine the role of Nox2 and Nox4 in angiogenesis, we employed Western blotting and immunofluorescence staining assays to assess the expression of Nox2 in placentae. The two groups showed no obvious difference in the mRNA expression of *Nox2 and Nox4* (Supplemental Fig. S1b and c) or in the protein expression level of Nox4 (Supplemental Fig. S1d and e). However, the protein expression level of Nox2 was higher in the LBW group than in the NBW group (Fig. 1i and j), and Nox2 was highly expressed in the vessels of LBW placentae (Fig. 1k and l). We also analyzed the correlation of Nox2 with CD31 expression and vascular density. The results showed that the Nox2 fluorescence intensity was negatively correlated with placental CD31 expression and vascular density (Fig. 1m and n). These above findings indicate that the increased Nox2 expression is related to poor angiogenesis in placentae.

### 3.2 Inhibition of Nox2 promotes angiogenesis

**Fig. 2:**
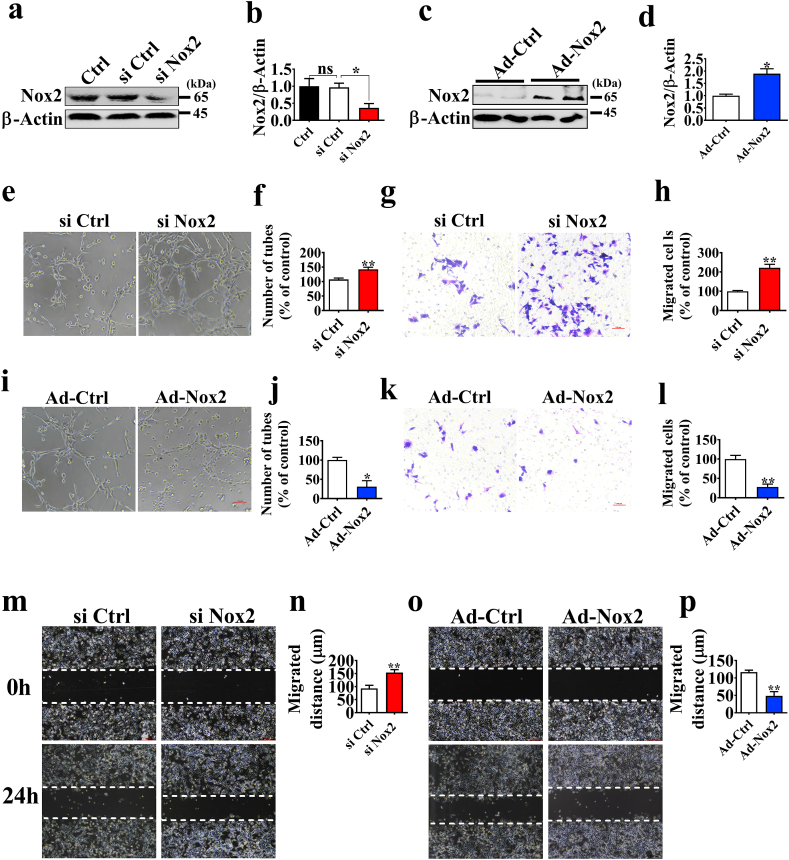
Nox2 regulates PVECs tube formation and migration. a-d. Western blotting analysis of Nox2 expression in PVECs with siRNA against or infected with adenovirus Nox2 for 48 h (n = 3). e, i. Representative images of tube formation by PVECs. PVECs were seeded at a density of 4 × 10^4^ cells/well on a plate precoated with Matrigel and cultured for 6 h after transfection with control siRNA or Nox2 siRNA, or with Ad-RFP or Ad-Nox2 for 48 h (n = 5). Scale bars, 200 μm. Images of Transwell (g, k) or wound healing assay (m, o) in each group (n = 5). Scale bars, 100 μm. All data represent the mean ± SEM. *P < 0.05, **P < 0.01, ***P < 0.001.

To test the hypothesis that the increased Nox2 expression is related to poor angiogenesis in placentae, we investigated whether Nox2 could influence angiogenesis *in vitro*. We established stable siRNA-Nox2 (si Nox2) and adenovirus-Nox2 (Ad-Nox2) expressing PVECs (Fig. 2a–d). We found that inhibition of Nox2 promoted endothelial cell tube formation and migration (Fig. 2e–h, m, n), whereas overexpression of Nox2 decreased endothelial cell tube formation and migration (Fig. 2i-l, o, p). Additionally, Nox2 ds-tat, a specific inhibitor of Nox2, was shown to increase PVECs tube formation and migration (Supplemental Fig. S2a-d).

### 3.3 VEGF-A mediates angiogenesis inhibition of Nox2 controlled

**Fig. 3:**
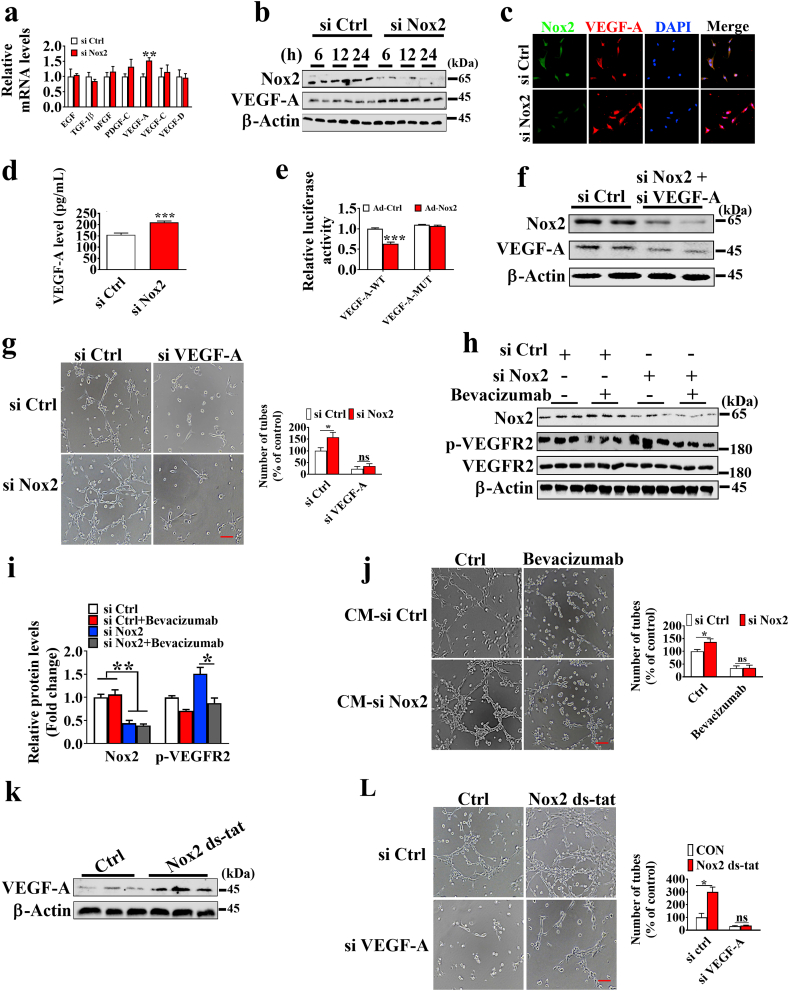
VEGF-A mediates the effects of Nox2 on PVECs tube formation. a. The mRNA expression of angiogenesis-related factors. Cells were transfected with indicated siRNA for 48 h, followed by culture for additional 24 h (n = 5). b. Western blotting analysis of the protein levels of Nox2 and VEGF-A in PVECs (n = 3). Cells were transfected with indicated siRNA for 48 h, followed by culture for additional 6, 12, and 24 h (n = 3). c. Immunofluorescence analysis of VEGF-A in PVECs. Cells were transfected with indicated siRNA for 48 h, followed by culture for additional 24 h. d. The concentration of VEGF-A in culture media (n = 5). e. Dual-luciferase reporter gene assay of the VEGF-A promoter activity (n = 5). f. Western blotting analysis of Nox2 and VEGF-A expression levels in PVECs. Cells were co-transfected with si Nox2 and si VEGF-A for 48 h (n = 6). g. The representative images of tube formation of PVECs. Cells were co-transfected with si Nox2 and si VEGF-A for 48 h and seeded at a density of 4 × 10^4^ cells/well on a plate precoated with Matrigel and cultured for 6 h (n = 5); scale bars, 200 μm. h, i. Western blotting analysis of Nox2 and p-VEGFR2 expression levels in PVECs with siRNA against Nox2 treated with or without bevacizumab (20 μg/mL) for 24 h. j. The representative images of tube formation of PVECs cultured with conditioned medium from the siRNA transfected cells treated with or without bevacizumab (20 μg/mL) for 6 h (n = 5); scale bars, 200 μm. k. The protein level of VEGF-A in PVECs under the treatment of Nox2 ds-tat (10 μM) (n = 3). l. The representative images of tube formation of PVECs with siRNA against VEGF-A treated with or without Nox2 ds-tat (10 μM) (n = 5); scale bars, 200 μm.

A real-time PCR was performed to determine the difference in the expression of angiogenesis-related factors in placentae and Nox2 knockdown cells. The results showed that the mRNA expression level of *VEGF-A* was higher in the NBW group than in the LBW group (Supplemental Fig. S3). Additionally, Nox2 knockdown significantly increased the mRNA expression level of *VEGF-A* (Fig. 3a). Western blotting and immunofluorescence analysis confirmed the increase of VEGF-A expression was observed in Nox2 knockdown cells (Fig. 3b and c). Furthermore, we examined the secretion of VEGF-A in the culture supernatants from si Ctrl and si Nox2 cells. The ELISA results indicated that the VEGF-A concentration increased in the conditioned media (CM) from si Nox2 cells versus si Ctrl cells (Fig. 3d). The overexpression of Nox2 in PVECs decreased the protein expression levels of VEGF-A (Supplemental Fig. S7). Moreover, the promoter activity of VEGF-A was also significantly reduced when PVECs were transfected with Nox2 adenovirus (Fig. 3e). Since Nox2 could inhibit PVECs tube formation and migration, we questioned whether VEGF-A-mediated angiogenesis inhibition is controlled by Nox2. Therefore, siRNA was used to knock down the expression of Nox2 and VEGF-A (Fig. 3f). After knockdown of VEGF-A, we found that no significant difference was observed in the tube formation between si Ctrl and si Nox2 groups (Fig. 3g), and knockdown of VEGF-A produced no effect on the protein expression level of Nox2 (Supplemental Fig. S4a). VEGF-neutralizing antibody, bevacizumab, was used to inhibit the VEGF-VEGFR2 signaling pathway (Fig. 3h), and no significant difference was observed in the tube formation and migration between si Ctrl and si Nox2 groups after bevacizumab treatment (Supplemental Fig. S4b, c). Additionally, we used CM to investigate whether Nox2-regulated VEGF-A expression stimulates autocrine angiogenesis. The CM from si Nox2 cells was found to have an increase in tube formation, while the CM derived from si Nox2 cells treated with bevacizumab showed no change in tube formation (Fig. 3j), implying that Nox2 knockdown induces VEGF-A dependent autocrine angiogenesis.

We also observed no significant difference in the tube formation between Ctrl and Nox2 ds-tat groups after knockdown of VEGF-A (Fig. 3k and l). Taken together, VEGF-A is the target of Nox2 during angiogenesis inhibition.

### 3.4 Nox2 indirectly regulates STAT3 signaling pathway

**Fig. 4:**
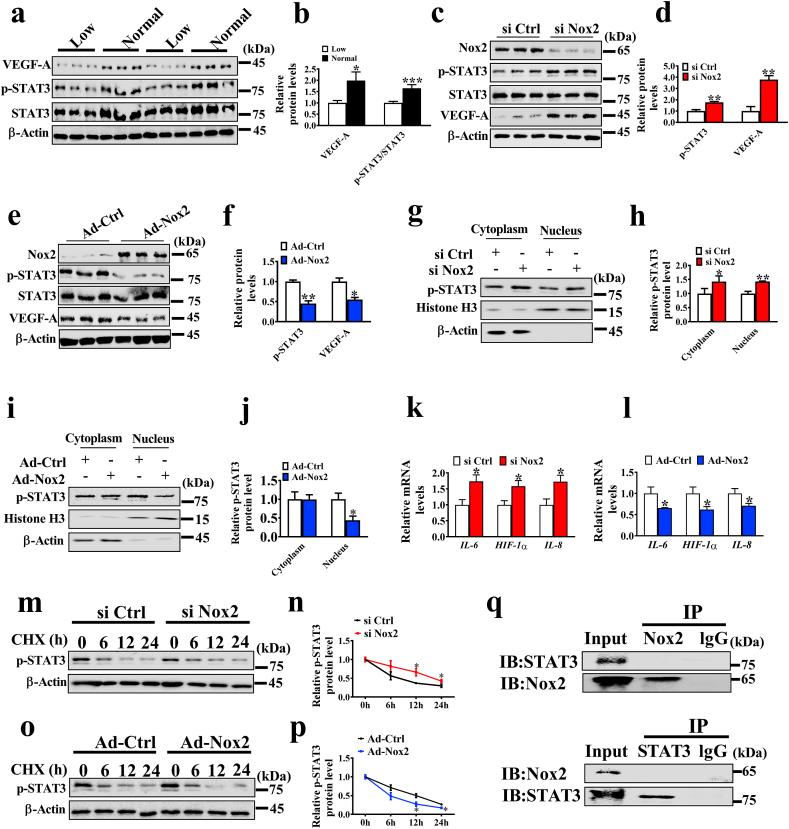
Nox2 regulates STAT3 signaling pathway. (a, b) The protein levels of VEGE-A, p-STAT3 and STAT3 in placentae (n = 12). c-f. Western blotting analysis of p-STAT3 and VEGF-A expression in control and Nox2-knockdown or Nox2-overexpression cells (n = 3). g-i. Western blotting analysis of nuclear and cytoplasmic distribution of p-STAT3 in PVECs with Nox2 knockdown or overexpression; histone H3 and β-Actin serve as nuclear and cytoplasmic markers, respectively (n = 3). k, l. The mRNA expression levels of STAT3-targeted genes (n = 5). m-p. Protein overturn assay. PVECs with Nox2 silence or overexpression were incubated with 12 μg/mL CHX for the indicated interval; Western blotting was employed to observe the p-STAT3 protein levels (n = 3). q. Immunoprecipitation (IP) analysis of the interaction of Nox2 and STAT3. *P < 0.05, **P < 0.01, ***P < 0.001.

To measure the kinetics of VEGF-A mRNA and protein degradation, we added actinomycin D (AD) and cycloheximide (CHX) to inhibit mRNA and protein synthesis, respectively. The si Ctrl and si Nox2 groups showed no significant difference in VEGF-A mRNA and protein degradation (Supplemental Fig. S5a-d). Additionally, transcription factors, including HIF-1α, NF-κB, and STAT3, were shown to regulate VEGF-A expression [[19], [20], [21]], and we used PX-478, QZN, and Stattic to inhibit HIF-1α, NF-κB, and STAT3, respectively. However, inhibition of HIF-1α and NF-κB failed to reverse the angiogenic effects induced by Nox2 knockdown, whereas inhibition of STAT3 phosphorylation produced no difference between si Ctrl and si Nox2 groups in tube formation (Supplemental Fig. S6). Meanwhile, the protein levels of VEGF-A and p-STAT3 were significantly decreased in the LBW group versus the NBW group (Fig. 4a and b). Western blotting revealed that the protein level of p-STAT3 was strongly up-regulated in the Nox2-knockdown PVECs (Fig. 4c and d), but down-regulated in the Nox2-overexpression PVECs (Fig. 4e and f). The nuclear and cytoplasmic proteins of the cells were collected for western blotting analysis, and the nuclear p-STAT3 expression was increased by Nox2 knockdown (Fig. 4g and h), but inhibited by Nox2 overexpression (Fig. 4i and j).

To further investigate the activation of STAT3, the mRNA expression of STAT3 downstream genes, including *IL-8*, *IL-6*, and *HIF-1α*, was determined by real-time -PCR in Nox2 knockdown cells or Nox2 overexpressing cells. The data showed that the mRNA levels of STAT3 target genes were increased in Nox2 knockdown cells, but decreased in Nox2 overexpressing cells (Fig. 4k and l). Additionally, to clarify the mechanism of how Nox2 regulates p-STAT3, we determined the effects of Nox2 on p-STAT3 protein stability, and the cell lysates were harvested at 0, 6, 12 and 24 h post CHX addition. The p-STAT3 protein was observed to degrade slower in si Nox2 cells than in si Ctrl cells, while faster in Nox2 overexpressing cells (Fig. 4m–p), indicating that Nox2 reduces the p-STAT3 stability. Nox2 knockdown was found to have no effect on STAT3 expression in PVECs treated with or without MG132 (Supplemental Fig. S8). Furthermore, whether Nox2 directly interacts with STAT3 was determined by co-immunoprecipitation (Co-IP). As shown in Fig. 4q, no direct interaction was observed between Nox2 and STAT3.

### 3.5 Nox2 reduces VEGF-A expression by STAT3

**Fig. 5:**
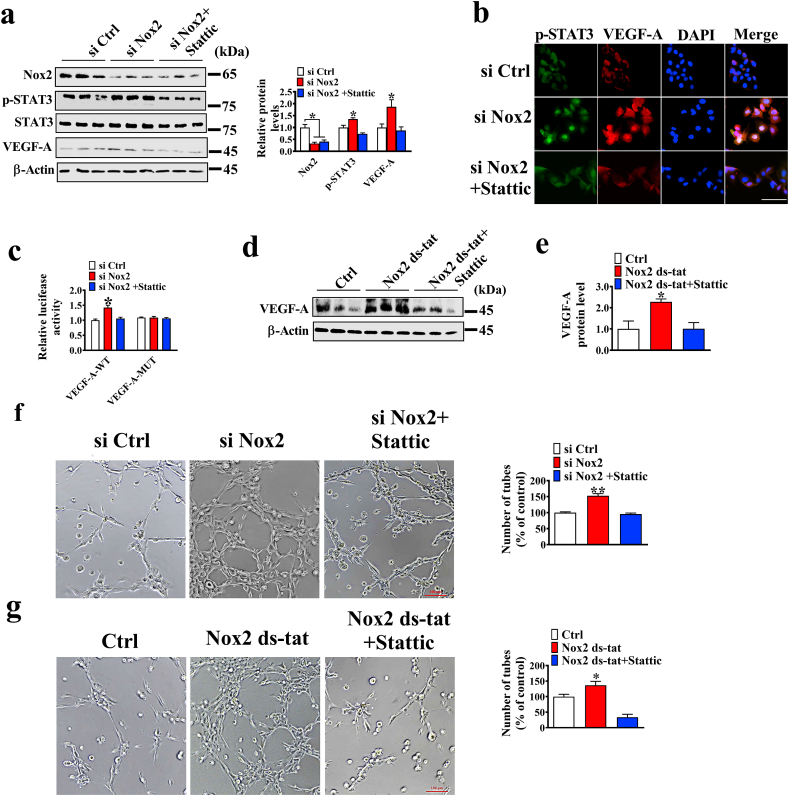
Nox2 regulates angiogenesis *via* activating the STAT3/VEGF-A signaling pathway. a. Western blotting analysis of Nox2, VEGF-A, and p-STAT3 protein levels in control and Nox2-knockdown cells treated with or without 5 μM Stattic (n = 3). b. Immunofluorescence analysis of VEGF-A and p-STAT3 in control and Nox2-knockdown cells treated with or without 5 μM Stattic. c. VEGF-A promoter activity (n = 5). d, e. Western blotting analysis of the VEGF-A protein level in PVECs (n = 3); cells were treated with 5 μM Stattic for 2 h, followed by stimulation with 10 μM Nox2 ds-tat for 24 h. f. The representative images of tube formation of PVECs transfected with si Ctrl or si Nox2 and treated with 5 μM Stattic for 6 h (n = 5); bar = 100 μm. g. The representative images of tube formation of PVECs treated with 10 μM Nox2 ds-tat and 5 μM Stattic (n = 5); bar = 100 μm.

Whether Nox2 knockdown-induced VEGF-A expression is dependent on STAT3 activation was investigated by treating the Nox2 knockdown cells with the STAT3 inhibitor Stattic. We found that STAT3 inhibition induced a decrease nearly to the level of the control group in VEGF-A protein and promoter activity (Fig. 5a–e). Furthermore, whether Nox2 knockdown-induced tube formation requires STAT3 activation was tested by blocking the STAT3 pathway. When STAT3 pathway was blocked, Nox2 knockdown or inhibition failed to facilitate PVECs tube formation (Fig. 5f–g)**.** These results revealed that Nox2 knockdown-induced VEGF-A expression depends on STAT3 activation.

### 3.6 Nox2 regulates p-STAT3 though mitochondrial ROS

**Fig. 6:**
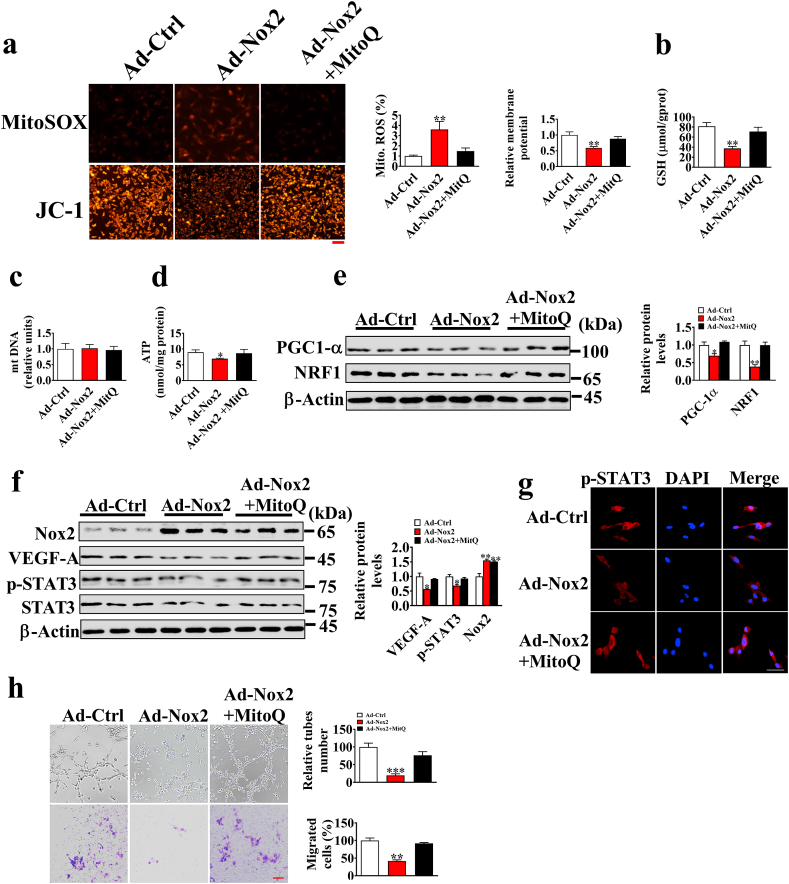
Nox2 overexpression inhibits the activation of the STAT3 signaling pathway by inducing mitochondrial ROS production. a. Representative fluorescence images of MitoSOX and JC-1-stained cells; scale bar: 100 μm. b-d. GSH, mt DNA, and ATP contents (n = 6). e. The protein levels of PGC-1α and NRF1 (n = 3). f. The Nox2, VEGF-A, and p-STAT3 protein levels of PVECs transfected with Ad-Ctrl or Ad- Nox2 under the treatment of MitoQ (500 nM) for 24 h (n = 3). g. Immunofluorescence analysis of p-STAT3 in PVECs transfected with Ad-Ctrl or Ad-Nox2 under the treatment of MitoQ for 24 h. h. Representative images of tube formation and transwell assay of PVECs (n = 5).

Co-IP results indicate that Nox2 may indirectly regulate p-STAT3 expression. Previous study has shown that mitochondrial ROS exerts an importance role in STAT3 activation [22]. Therefore, we detected the mitochondrial ROS levels using MitoSOX. As shown in Fig. 6, Nox2 overexpression could significantly increase the mitochondrial ROS levels, whereas Nox2 knockdown could reduce the formation of ROS and mitochondrial ROS (Supplemental Fig. S9). Additionally, membrane potential, GSH, and ATP levels were also decreased in PVECs (Fig. 6a–d), indicating Nox2 overexpression causes mitochondrial dysfunction [23]. PGC1-α and NRF1 are regulators of mitochondrial biogenesis, and their protein levels were also decreased in Nox2 overexpression cells (Fig. 6e). Furthermore, we employed MitoQ (a mitochondrial ROS scavenger) to obliterate mitochondrial ROS, thereby restoring the protein expression levels of p-STAT3 and VEGF-A to normal levels (Fig. 6f, g), and a similar result was observed in the tube formation and migration of PVECs (Fig. 6h). These results indicate that Nox2 regulates STAT3/VEGF-A pathway via mitochondrial ROS.

### 3.7 Knockdown of Nox2 increases angiogenesis in matrigel plugs *in vivo*

**Fig. 7:**
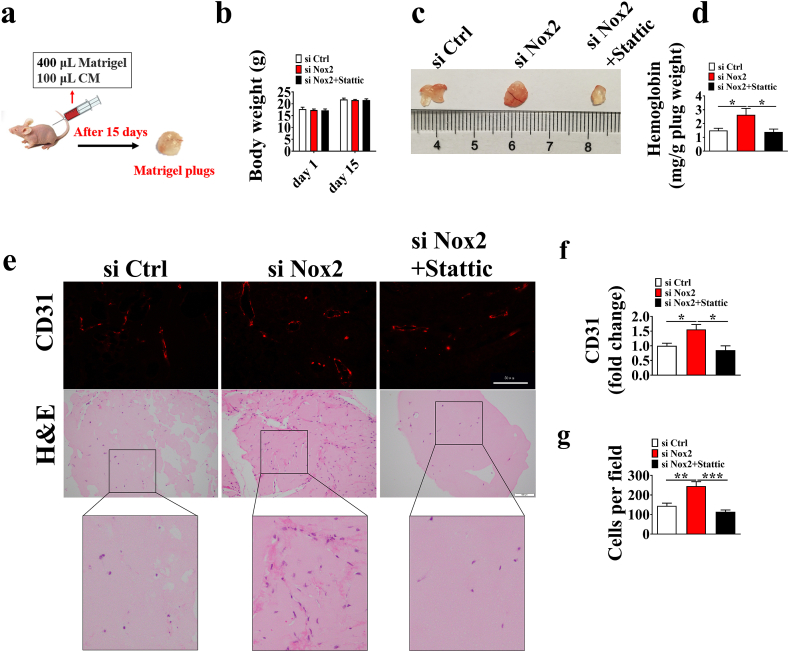
Knockdown of Nox2 induces angiogenesis in Matrigel plugs *in vivo*. a. BALB/c nude mice were injected subcutaneously with 400 μL Matrigel mixed with 100 μL PVEC conditioned media (CM) for 15 days, then the plugs were excised. b. Body weight of BALB/c nude mice at day 1 and 15 in the trial. c. The Matrigel plugs were photographed (c), quantified for hemoglobin content (d), stained with CD31(e, upper) and hematoxylin and eosin (e, below). The CD31 immunofluorescence intensity (f) and cells (g) in the Matrigel plugs. Results are expressed as the mean ± SEM (n = 6).

**Fig. 8:**
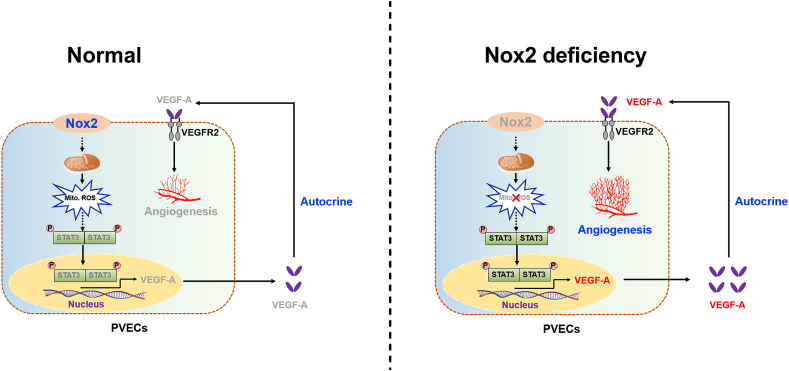
Schematic diagram showing the role of Nox2-mediated VEGF-A dependent angiogenesis via STAT3 signaling. Nox2 deficiency decreased mitochondrial ROS generation, which in turn induced STAT3 activation, thereby increasing VEGF-A expression and excretion and promoting VEGF-A dependent autocrine angiogenesis.

We used the Matrigel plug assay to evaluate the effects of Nox2 on angiogenesis (Fig. 7a). No difference was observed in the body weight among si Ctrl, si Nox2, and si Nox2+stattic groups (Fig. 7b), and the hemoglobin levels were increased by knockdown of Nox2 (Fig. 7c and d). CD31 and H&E staining revealed that Matrigel mixed with CM from the si Nox2 group increased the micro-vessel formation and contained more cells in the Matrigel plugs (Fig. 7e–g). To assess STAT3/VEGF-A dependent autocrine angiogenesis *in vivo*, the BALB/c nude mice were given subcutaneous injections of Matrigel containing CM from si Ctrl or si Nox2 cells treated with or without STAT3 inhibitor (Stattic). Under the treatment of STAT3 inhibitor Stattic, Nox2 knockdown showed a decrease nearly to the levels of the si Ctrl group in hemoglobin, CD31 immunofluorescence intensity, and cells in the Matrigel plugs (Fig. 7e–g). These results indicate that Nox2 knockdown induces angiogenesis *in vivo*. A schematic diagram for the role of Nox2-mediated VEGF-A dependent angiogenesis *via* STAT3 signaling is shown in Fig. 8.

## 4 Discussion

Previous studies have documented that Nox2 is associated with angiogenesis [24]. However, the functional roles of Nox2 in regulating placental angiogenesis remain unclear. In this study, we demonstrated that LBW placentae showed low placental vessel density, which was consistent with the finding of a previous study [5]. Studies have also shown that high oxidative stress induced the dysfunction of endothelial cells, thus contributing to aberrant angiogenesis [25]. Here, we found a higher oxidative damage in placentae for LBW fetuses, as evidenced by the higher ROS and MDA levels. This finding was in line with a previous report of a higher oxidative damage in placentae for IUGR fetuses [26]. Nox2 and Nox4 are shown to be the main sources of ROS in endothelial cells [27]. Interestingly, the LBW group showed no difference from the NBW group in the mRNA levels of Nox2 and Nox4 in placentae, but was significantly higher than the NBW group in the protein level of Nox2. The expression level of protein depends on the balance between their synthesis and degradation, suggesting that Nox2 might have a lower rate of degradation and/or a higher rate of synthesis in the LBW group than in the NBW group. MicroRNAs (miRNAs) are important regulators of gene expression by modulating translation [28]. miRNAs, such as miRNA-141, miRNA-106b, miRNA-148b, and miRNA-204, were identified to regulate Nox2 expression [29,30], indicating that specific miRNAs might be involved in the regulation of Nox2 on protein expression in placentae.

Nox2 deficiency was reported to protect against the hypercholesterolemia-induced impairment of neovascularization by reducing ROS formation, improving the functional activity of endothelial progenitor cells, and maturing endothelial cells [13]. Therefore, we suspect that the increased Nox2 level in placenta might induce excessive ROS generation, contributing to the decrease of angiogenesis. To investigate the role of Nox2 in angiogenesis, we established stable Nox2 knockdown and overexpression in PVECs. As expected, knockdown of Nox2 in PVECs significantly promoted tube formation and migration, whereas overexpression of Nox2 inhibited tube formation and migration *in vitro*. Previous results also confirmed that impairment of bone vessels was effectively ameliorated by treatment with Nox2 inhibitors [31]. These findings demonstrate the potential of Nox2 overactivation to decrease placental angiogenesis. Meanwhile, there was also evidence supporting that activation of Nox2 triggers angiogenesis [24,27]. This difference can be explained by the physiological and pathological angiogenesis orchestrated by Nox2. In physiological angiogenesis, Nox2 overactivation could induce excessive ROS generation, thus impairing the function of vascular endothelial cells [32]. In pathological angiogenesis, high levels of ROS induced by Nox2 may damage DNA, leading to genomic instability and activation of proteins, such as NF-κB and HIF-1α, which are known to promote pathological angiogenesis [33,34].

Angiogenesis is regulated by angiogenic factors, such as VEGF, TGF-1β, PDGF, bFGF, and EGF [19]. To explore the target of Nox2 for inhibiting angiogenesis, a real-time PCR was performed and knockdown of Nox2 was found to promote the mRNA expression of VEGF-A. Meanwhile, the LBW group showed a significant downregulation in the mRNA and protein expression of VEGF-A. This finding suggested the involvement of VEGF-A in the role of Nox2 in placental angiogenesis. VEGF-A is the main mediator of angiogenesis in endothelial cells, which induces endothelial cell proliferation and migration to form neovascular [35]. Several VEGF-targeted therapies, especially Bevacizumab, were widely used in antiangiogenic treatment. Here, we demonstrated that the effect of Nox2 knockdown or inhibition on PVECs angiogenesis could be reversed by interference with VEGF-A *in vitro*, which was in line with a previous report that knockdown or inhibition of VEGF-A impairs angiogenesis *in vitro* [19]*.* In addition, our results indicate that Nox2 knockdown induces VEGF-A dependent autocrine angiogenesis, which was well supported by previous findings that boosting an autocrine VEGF-VEGFR2 positive-feedback signaling loop led to amplified effect of VEGF on angiogenesis, proliferation, and migration *in vitro* and *in vivo* [36].

The expression and secretion of VEGF-A are regulated by transcription factors, and STAT3 is known to activate VEGF-A and promote its secretion in cells [20]. Additionally, activation of STAT3 was shown to regulate cell proliferation, migration, and angiogenesis [37]. Thus, we examined the changes in the STAT3 signaling pathway in PVECs by inhibition or overexpression of Nox2. Nox2 inhibition and overexpression were found to markedly increase and decrease the phosphorylation of STAT3 and nuclear localization in cells, respectively. Moreover, the LBW group was lower than the NBW group in the p-STAT3 protein level. IL-6, HIF-1α, and IL-8 are the downstream genes of STAT3 [38], and consistent with the changes of p-STAT3, the mRNA levels of these downstream genes were up-regulated in cells when Nox2 was inhibited, further indicating that Nox2 inhibition induced the activation of STAT3. Small GTP-binding protein Rac1, one of the members of Nox2 holoenzyme, might be involved in the activation of STAT3 induced by Nox2. A previous study mentioned that Rac1 induced the phosphorylation of STAT3 on Ser-727 by activation of JNK or an indirect mechanism involving the action of IL-6 [39]. Interestingly, we found a slower degradation of p-STAT3 protein in the Nox2-knockdown cells than in the control cells. The ubiquitin-proteasomal degradation is one of major pathways for regulating protein stability. In a previous study, polyubiquitylation and proteasomal modification were found to reduce p-STAT3 degradation, thus contributing to increased p-STAT3 stability and angiogenesis [20]. These results suggest that Nox2-knockdown could increase the stability of p-STAT3 protein *via* disrupting its polyubiquitination and proteasomal proteolysis system.

Inhibiting the phosphorylation of STAT3 could completely reverse the effect of Nox2 knockdown on VEGF-A expression and angiogenesis, which was in line with a previous report of VEGF-independent angiogenesis through STAT3 signaling [17]. These results demonstrate that Nox2 knockdown induces VEGF-A mediated autocrine angiogenesis *via* STAT3 *in vitro*. To address the role of STAT3 in VEGF-A dependent autocrine angiogenesis *in vivo*, the BALB/c nude mice were given injections of CM from Nox2 knockdown cells treated with STAT3 inhibitor. We found that Nox2 knockdown failed to promote angiogenesis and cell migration under the treatment of STAT3 inhibitor *in vivo*, which is consistent with our hypothesis that STAT3 inhibition could decrease Nox2 knockdown-induced VEGF-A expression, thus contributing to the decrease of angiogenesis and migration. Consistent with our study, a previous study stated that an autocrine VEGF signaling is required for vascular homeostasis [40]. Taken together, our results support that STAT3/VEGF-A plays a central role in Nox2 inhibition of angiogenesis *in vitro* and *in vivo*.

Many questions remain to be further clarified. In the present study, Nox2 overexpression was shown to decrease STAT3 phosphorylation and nuclear localization in cells, thereby decreasing the expression of STAT3-targeting genes including VEGF-A (Supplemental Fig. S4). However, the mechanism by which Nox2 regulates STAT3 phosphorylation remains to be elucidated. Meanwhile, immunoprecipitation analysis showed no interaction between Nox2 and STAT3, indicating Nox2 regulates STAT3 in an indirect way. Mitochondrial ROS accumulation has been reported to inhibit the STAT3 pathway [22], and Nox2 is shown to exist in intracellular compartments or plasma membranes and induce the production of mitochondrial ROS by activating reverse electron transfer, and the enhanced production of mitochondrial ROS further activates the cytoplasmic NADPH oxidases, thus increasing cellular O^2−^ production [[41], [42], [43]]. Rac1 also mediated the production of mitochondrial ROS through Nox2 [44,45], and the increased mitochondrial ROS inhibited the activation of JAK kinase (an upstream kinase of STAT3), thus inhibiting the activation of STAT3 [22,46]. Therefore, we suspected that Nox2 overexpression induces excessive mitochondrial ROS generation to inhibit the STAT3 pathway. Here, Nox2 overexpression was observed to increase mitochondrial ROS generation, and the inhibitory effect of Nox2 overexpression on STAT3 pathway and angiogenesis was reversed by obliterating mitochondrial ROS. These results suggest that Nox2 is an important contributor to excessive mitochondrial ROS production in PVECs and impairment of STAT3 activation and angiogenic functions.

## 5 Conclusions

In summary, this is the first demonstration that Nox2 is highly expressed in LBW placenta and reduces placental angiogenesis *via* a mitochondrial ROS-STAT3-VEGF-A dependent mechanism (Fig. 8). This study provides insights into the underlying molecular mechanisms of Nox2 in regulating placental angiogenesis and facilitates the development of therapeutic strategies for IUGR prevention.

## Declaration of competing interest

The authors declare that they have no conflict of interest.

## Appendix A Supplementary data

Application Application

## References

[1] Longo S., Bollani L., Decembrino L., Di Comite A., Angelini M., Stronati M. (2013). Short-term and long-term sequelae in intrauterine growth retardation (IUGR). J. Matern. Fetal Neonatal Med..

[2] Wu G., Bazer F.W., Cudd T.A., Meininger C.J., Spencer T.E. (2004). Maternal nutrition and fetal development. J. Nutr..

[3] Zur R.L., Kingdom J.C., Parks W.T., Hobson S.R. (2020). The placental basis of fetal growth restriction. Obstet. Gynecol. Clin. N. Am..

[4] Su E.J., Xin H., Yin P., Dyson M., Coon J., Farrow K.N., Mestan K.K., Ernst L.M. (2015). Impaired fetoplacental angiogenesis in growth-restricted fetuses with abnormal umbilical artery Doppler velocimetry is mediated by aryl hydrocarbon receptor nuclear translocator (ARNT). J. Clin. Endocrinol. Metab..

[5] Song T., Lu J., Deng Z., Xu T., Yang Y., Wei H., Li S., Jiang S., Peng J. (2018). Maternal obesity aggravates the abnormality of porcine placenta by increasing N(6)-methyladenosine. Int. J. Obes..

[6] Carr D.J., David A.L., Aitken R.P., Milne J.S., Borowicz P.P., Wallace J.M., Redmer D.A. (2016). Placental vascularity and markers of angiogenesis in relation to prenatal growth status in overnourished adolescent ewes. Placenta.

[7] Nowak-Sliwinska P., Alitalo K., Allen E., Anisimov A., Aplin A.C., Auerbach R., Augustin H.G., Bates D.O., van Beijnum J.R., Bender R.H.F., Bergers G., Bikfalvi A., Bischoff J., Böck B.C., Brooks P.C., Bussolino F., Cakir B., Carmeliet P., Castranova D., Cimpean A.M., Cleaver O., Coukos G., Davis G.E., De Palma M., Dimberg A., Dings R.P.M., Djonov V., Dudley A.C., Dufton N.P., Fendt S.M., Ferrara N., Fruttiger M., Fukumura D., Ghesquière B., Gong Y., Griffin R.J., Harris A.L., Hughes C.C.W., Hultgren N.W., Iruela-Arispe M.L., Irving M., Jain R.K., Kalluri R., Kalucka J., Kerbel R.S., Kitajewski J., Klaassen I., Kleinmann H.K., Koolwijk P., Kuczynski E., Kwak B.R., Marien K., Melero-Martin J.M., Munn L.L., Nicosia R.F., Noel A., Nurro J., Olsson A.K., Petrova T.V., Pietras K., Pili R., Pollard J.W., Post M.J., Quax P.H.A., Rabinovich G.A., Raica M., Randi A.M., Ribatti D., Ruegg C., Schlingemann R.O., Schulte-Merker S., Smith L.E.H., Song J.W., Stacker S.A., Stalin J., Stratman A.N., Van de Velde M., van Hinsbergh V.W.M., Vermeulen P.B., Waltenberger J., Weinstein B.M., Xin H., Yetkin-Arik B., Yla-Herttuala S., Yoder M.C., Griffioen A.W. (2018). Consensus guidelines for the use and interpretation of angiogenesis assays. Angiogenesis.

[8] Heinolainen K., Karaman S., D'Amico G., Tammela T., Sormunen R., Eklund L., Alitalo K., Zarkada G. (2017). VEGFR3 modulates vascular permeability by controlling VEGF/VEGFR2 signaling. Circ. Res..

[9] Hu C., Yang Y., Deng M., Yang L., Shu G., Jiang Q., Zhang S., Li X., Yin Y. (2020). Placentae for low birth weight piglets are vulnerable to oxidative stress, mitochondrial dysfunction, and impaired angiogenesis. Oxid. Med. Cell Longev..

[10] Hu X.F., Wang L., Xiang G., Lei W., Feng Y.F. (2018). Angiogenesis impairment by the NADPH oxidase-triggered oxidative stress at the bone-implant interface: critical mechanisms and therapeutic targets for implant failure under hyperglycemic conditions in diabetes. Acta Biomater..

[11] Yin H., Xu L., Porter N.A. (2011). Free radical lipid peroxidation: mechanisms and analysis. Chem. Rev..

[12] Hahner F., Moll F., Schröder K. (2020). NADPH oxidases in the differentiation of endothelial cells. Cardiovasc. Res..

[13] Haddad P., Dussault S., Groleau J., Turgeon J., Maingrette F., Rivard A. (2011). Nox2-derived reactive oxygen species contribute to hypercholesterolemia-induced inhibition of neovascularization: effects on endothelial progenitor cells and mature endothelial cells. Atherosclerosis.

[14] Turgeon J., Haddad P., Dussault S., Groleau J., Maingrette F., Perez G., Rivard A. (2012). Protection against vascular aging in Nox2-deficient mice: impact on endothelial progenitor cells and reparative neovascularization. Atherosclerosis.

[15] Hu C., Yang Y., Li J., Wang H., Cheng C., Yang L., Li Q., Deng J., Liang Z., Yin Y., Xie Z., Tan C. (2019). Maternal diet-induced obesity compromises oxidative stress status and angiogenesis in the porcine placenta by upregulating Nox2 expression. Oxid. Med. Cell Longev..

[16] Zhang Y.H., Wang D.W., Xu S.F., Zhang S., Fan Y.G., Yang Y.Y., Guo S.Q., Wang S., Guo T., Wang Z.Y., Guo C. (2018). α-Lipoic acid improves abnormal behavior by mitigation of oxidative stress, inflammation, ferroptosis, and tauopathy in P301S Tau transgenic mice. Redox Biol.

[17] Du Y.E., Tu G., Yang G., Li G., Yang D., Lang L., Xi L., Sun K., Chen Y., Shu K., Liao H., Liu M., Hou Y. (2017). MiR-205/YAP1 in activated fibroblasts of breast tumor promotes VEGF-independent angiogenesis through STAT3 signaling. Theranostics.

[18] Henríquez-Olguin C., Knudsen J.R. (2019). Cytosolic ROS production by NADPH oxidase 2 regulates muscle glucose uptake during exercise. Nat. Commun..

[19] Dai L., Cui X., Zhang X., Cheng L., Liu Y., Yang Y., Fan P., Wang Q., Lin Y., Zhang J., Li C., Mao Y., Wang Q., Su X., Zhang S., Peng Y., Yang H., Hu X., Yang J., Huang M., Xiang R., Yu D., Zhou Z., Wei Y., Deng H. (2016). SARI inhibits angiogenesis and tumour growth of human colon cancer through directly targeting ceruloplasmin. Nat. Commun..

[20] Zhao J., Du P., Cui P., Qin Y., Hu C., Wu J., Zhou Z., Zhang W., Qin L., Huang G. (2018). LncRNA PVT1 promotes angiogenesis via activating the STAT3/VEGFA axis in gastric cancer. Oncogene.

[21] Liu J.Y., Zeng Q.H., Cao P.G., Xie D., Chen X., Yang F., He L.Y., Dai Y.B., Li J.J., Liu X.M., Zeng H.L., Zhu Y.X., Gong L., Cheng Y., Zhou J.D., Hu J., Bo H., Xu Z.Z., Cao K. (2018). RIPK4 promotes bladder urothelial carcinoma cell aggressiveness by upregulating VEGF-A through the NF-κB pathway. Br. J. Canc..

[22] Cao Y., Wang J., Tian H., Fu G.H. (2020). Mitochondrial ROS accumulation inhibiting JAK2/STAT3 pathway is a critical modulator of CYT997-induced autophagy and apoptosis in gastric cancer. J. Exp. Clin. Canc. Res..

[23] Chen Q., Wang N., Zhu M., Lu J., Zhong H., Xue X., Guo S., Li M., Wei X., Tao Y., Yin H. (2018). TiO(2) nanoparticles cause mitochondrial dysfunction, activate inflammatory responses, and attenuate phagocytosis in macrophages: a proteomic and metabolomic insight. Redox Biol.

[24] Vara D., Watt J.M., Fortunato T.M., Mellor H., Burgess M., Wicks K., Mace K., Reeksting S., Lubben A., Wheeler-Jones C.P.D., Pula G. (2018). Direct activation of NADPH oxidase 2 by 2-Deoxyribose-1-Phosphate triggers nuclear factor kappa B-dependent angiogenesis. Antioxidants Redox Signal..

[25] Kim Y.W., Byzova T.V. (2014). Oxidative stress in angiogenesis and vascular disease. Blood.

[26] Schneider D., Hernández C., Farías M., Uauy R., Krause B.J., Casanello P. (2015). Oxidative stress as common trait of endothelial dysfunction in chorionic arteries from fetuses with IUGR and LGA. Placenta.

[27] Petry A., Djordjevic T., Weitnauer M., Kietzmann T., Hess J., Görlach A. (2006). NOX2 and NOX4 mediate proliferative response in endothelial cells, Antioxid. Redox Signal.

[28] Selbach M., Schwanhäusser B., Thierfelder N., Fang Z., Khanin R., Rajewsky N. (2008). Widespread changes in protein synthesis induced by microRNAs. Nature.

[29] Quan B., Zhang H., Xue R. (2019). miR-141 alleviates LPS-induced inflammation injury in WI-38 fibroblasts by up-regulation of NOX2. Life Sci..

[30] Yang J., Brown M.E., Zhang H., Martinez M., Zhao Z., Bhutani S., Yin S., Trac D., Xi J.J., Davis M.E. (2017). High-throughput screening identifies microRNAs that target Nox2 and improve function after acute myocardial infarction. Am. J. Physiol. Heart Circ. Physiol..

[31] Hu X.F., Xiang G., Wang T.J., Ma Y.B., Zhang Y., Yan Y.B., Zhao X., Wu Z.X., Feng Y.F., Lei W. (2021). Impairment of type H vessels by NOX2-mediated endothelial oxidative stress: critical mechanisms and therapeutic targets for bone fragility in streptozotocin-induced type 1 diabetic mice. Theranostics.

[32] Li Y., Cifuentes-Pagano E., DeVallance E.R., de Jesus D.S., Sahoo S., Meijles D.N., Koes D., Camacho C.J., Ross M., St Croix C., Pagano P.J. (2019). NADPH oxidase 2 inhibitors CPP11G and CPP11H attenuate endothelial cell inflammation & vessel dysfunction and restore mouse hind-limb flow. Redox Biol.

[33] Jackson A.L., Loeb L.A. (2001). The contribution of endogenous sources of DNA damage to the multiple mutations in cancer. Mutat. Res..

[34] Yang Z., Huang Y., Zhu L., Yang K., Liang K., Tan J., Yu B. (2021). SIRT6 promotes angiogenesis and hemorrhage of carotid plaque via regulating HIF-1α and reactive oxygen species. Cell Death Dis..

[35] Staels W., Heremans Y., Heimberg H., De Leu N. (2019). VEGF-A and blood vessels: a beta cell perspective. Diabetologia.

[36] Song F., Chen Q., Rao W., Zhang R., Wang Y., Ge H., Wei Q. (2019). OVA66 promotes tumour angiogenesis and progression through enhancing autocrine VEGF-VEGFR2 signalling. EBioMedicine.

[37] Hu F., Sun X., Li G., Wu Q., Chen Y., Yang X., Luo X., Hu J., Wang G. (2018). Inhibition of SIRT2 limits tumour angiogenesis via inactivation of the STAT3/VEGFA signalling pathway. Cell Death Dis..

[38] Chen R.Y., Yen C.J., Liu Y.W., Guo C.G., Weng C.Y., Lai C.H., Wang J.M., Lin Y.J., Hung L.Y. (2020). CPAP promotes angiogenesis and metastasis by enhancing STAT3 activity. Cell Death Differ..

[39] Faruqi T.R., Gomez D., Bustelo X.R., Bar-Sagi D., Reich N.C. (2001). Rac1 mediates STAT3 activation by autocrine IL-6. Proc. Natl. Acad. Sci. U S A..

[40] Lee S., Chen T.T., Barber C.L., Jordan M.C., Murdock J., Desai S., Ferrara N., Nagy A., Roos K.P., Iruela-Arispe M.L. (2007). Autocrine VEGF signaling is required for vascular homeostasis. Cell.

[41] Dikalov S.I., Nazarewicz R.R., Bikineyeva A., Hilenski L., Lassègue B., Griendling K.K., Harrison D.G., Dikalova A.E. (2014). Nox2-induced production of mitochondrial superoxide in angiotensin II-mediated endothelial oxidative stress and hypertension. Antioxidants Redox Signal..

[42] Kim Y.M., Kim S.J., Tatsunami R., Yamamura H., Fukai T., Ushio-Fukai M. (2017). ROS-induced ROS release orchestrated by Nox4, Nox2, and mitochondria in VEGF signaling and angiogenesis. Am. J. Physiol. Cell Physiol..

[43] Ushio-Fukai M. (2009). Compartmentalization of redox signaling through NADPH oxidase-derived ROS. Antioxidants Redox Signal..

[44] Acevedo A., González-Billault C. (2018). Crosstalk between Rac1-mediated actin regulation and ROS production. Free Radic. Biol. Med..

[45] Osborn-Heaford H.L., Murthy S., Gu L., LarsonCasey J.L., Ryan A.J., Shi L., Glogauer M., Neighbors J.D., Hohl R., Carter A.B. (2015). Targeting the isoprenoid pathway to abrogate progression of pulmonary fibrosis. Free Radic. Biol. Med..

[46] Monroe R.K., Halvorsen S.W. (2009). Environmental toxicants inhibit neuronal Jak tyrosine kinase by mitochondrial disruption. Neurotoxicology.

