# Supplementary material for: Nox2 impairs VEGF-A-induced angiogenesis in placenta via mitochondrial ROS-STAT3 pathway
Source: Redox Biol. 2021 Jun 18;45:102051. doi: 10.1016/j.redox.2021.102051 (PMC8258686; doi:10.1016/j.redox.2021.102051)
Supplement: Application [file mmc1.docx]

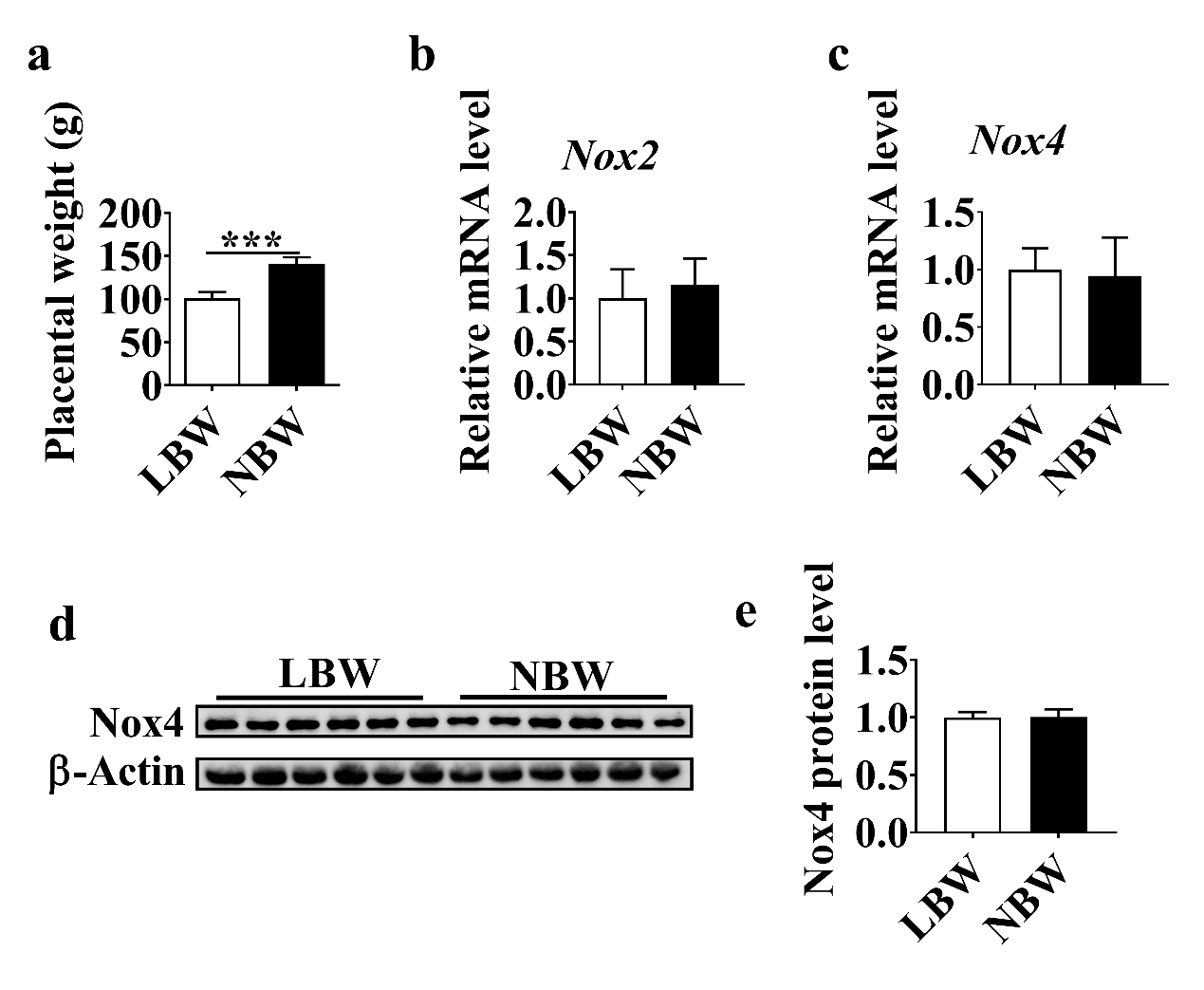


Supplemental Fig. S1. The placental weight (a) and expression levels of *Nox2* (b) and *Nox4* (c-e) in placentae. LBW, low birth weight group; NBW, normal birth weight group. ****P* < 0.001.


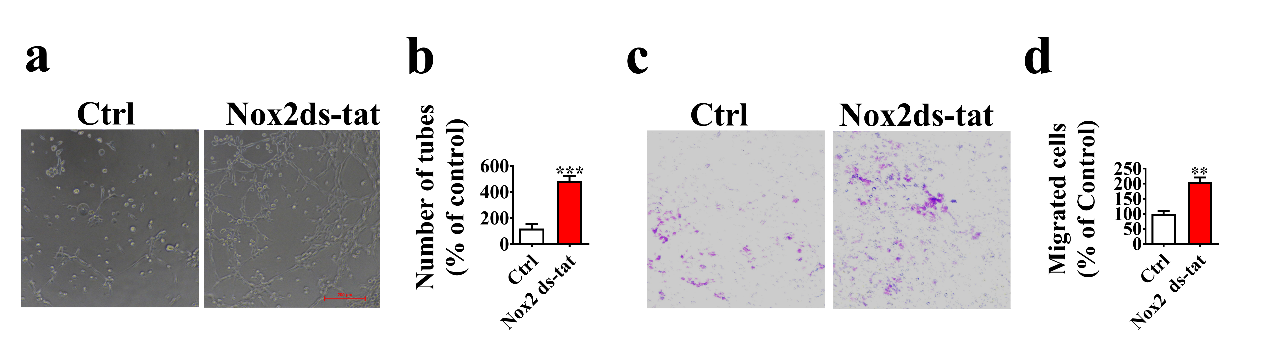


Supplemental Fig. S2. Inhibition of Nox2 promotes angiogenesis. PVECs treated with or without 10 μM Nox2 ds-tat for 24 h and seeded at a density of 4×10^4^ cells/well on a plate precoated with Matrigel or seeded into the upper chamber (n=5).


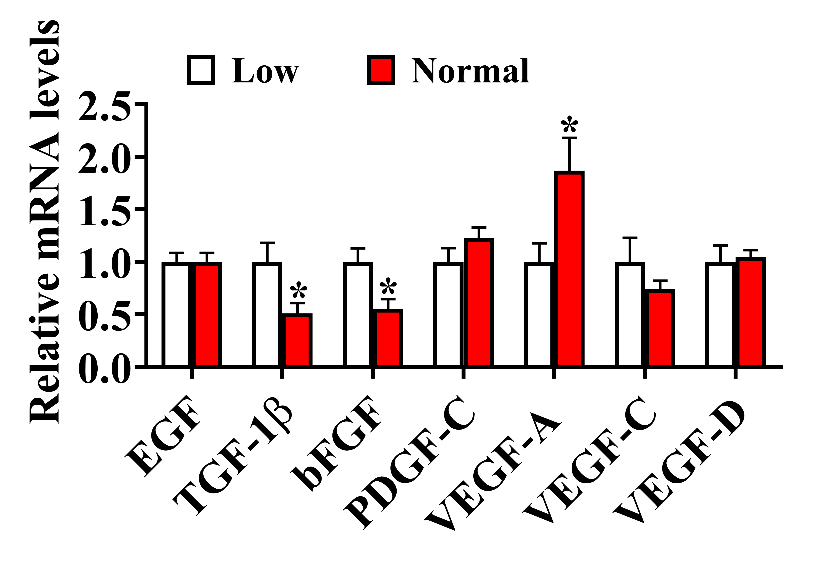


Supplemental Fig. S3. The mRNA expression levels of angiogenic factors in placenta.


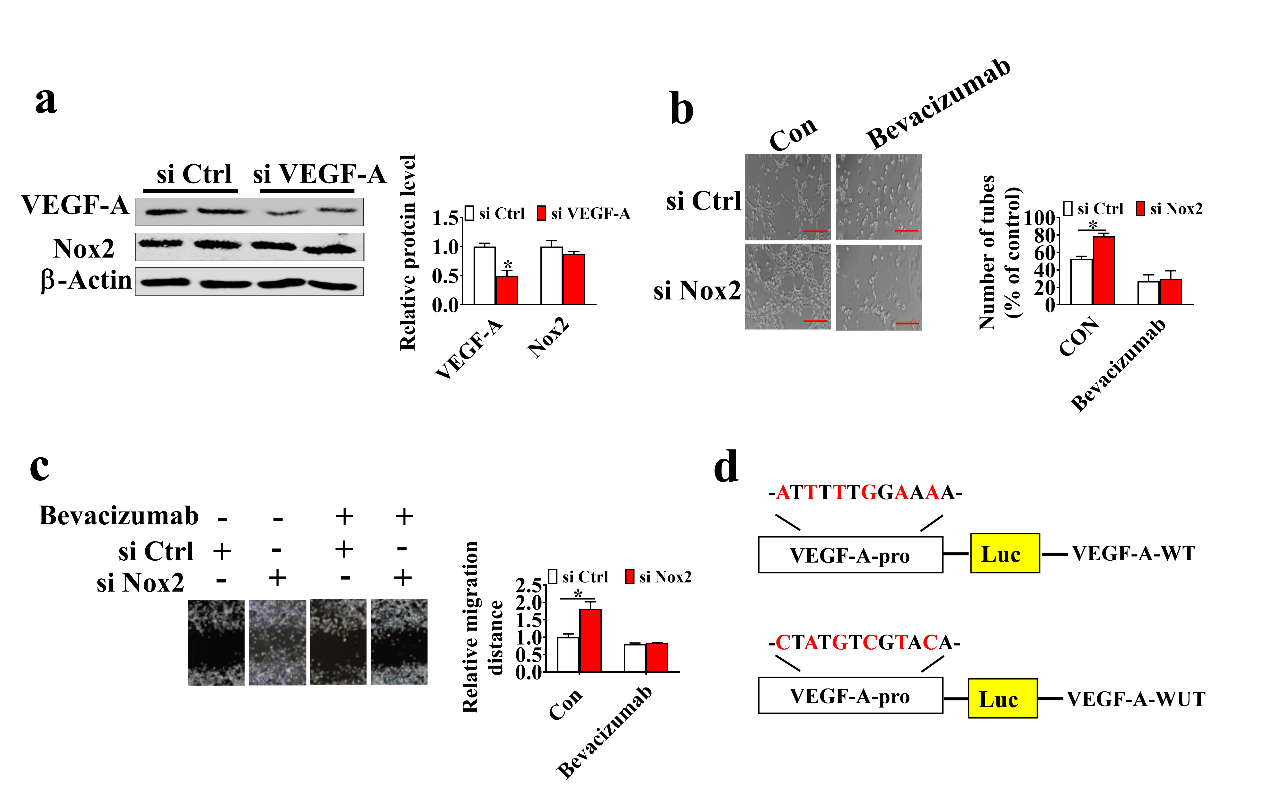


Supplemental Fig. S4. VEGF-A mediates the effects of Nox2 on PVECs tube formation. a. Western blotting analysis of VEGF-A and Nox2 protein levels in control and VEGF-A-knockdown cells. b. The representative images of tube formation of siRNA transfected cells treated with or without bevacizumab (20 μg/mL) for 6 h (n=5); scale bars: 200 μm. c. Images of wound healing assay in each group (n=5); scale bars:100 μm. All data represent the mean±SEM. **P*<0.05. d. Schematic diagram of dual luciferase reporter constructs.


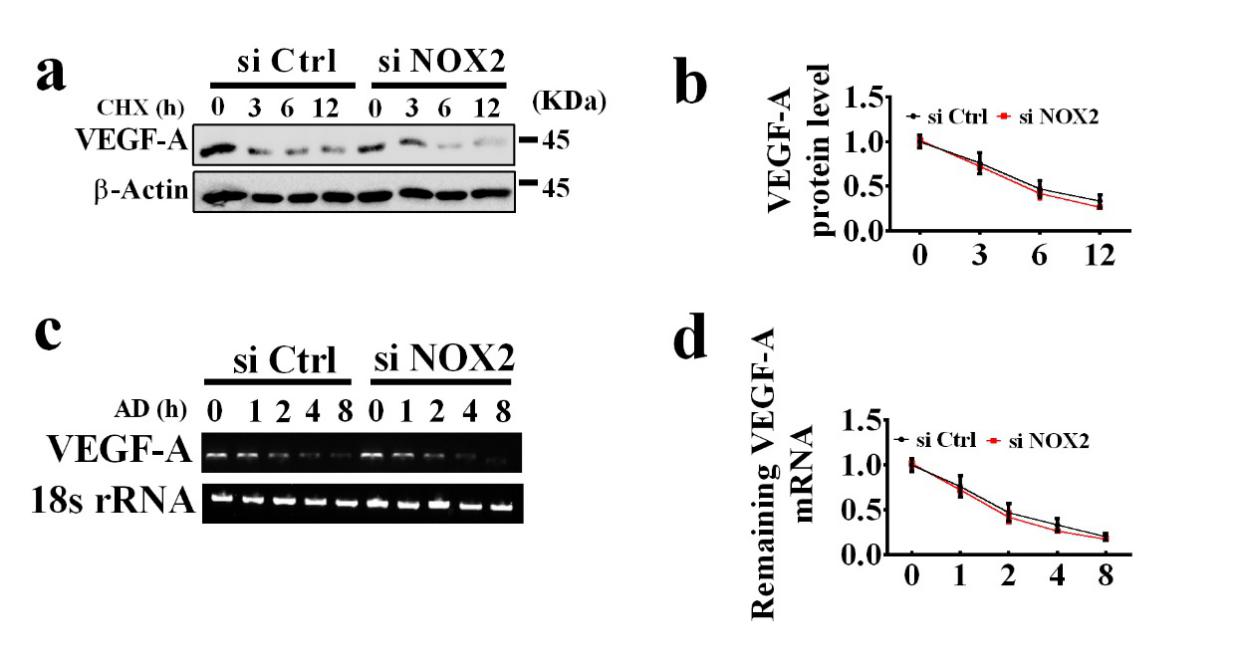


Supplemental Fig. S5. Knockdown of Nox2 had no effect on VEGF-A protein or mRNA stability. a. Protein overturn assay. PVECs with Nox2 silence were incubated with 12 μg/mL cycloheximide (CHX) for the indicated intervals. Western blotting was employed to observe the VEGF-A protein levels. (n=3). b. PVECs were transfected with si Ctrl or si Nox2 for 48 h, then treated with 10 μg/mL actinomycin D for the indicated intervals. VEGF-A mRNA levels were analyzed by qPCR (n=3).


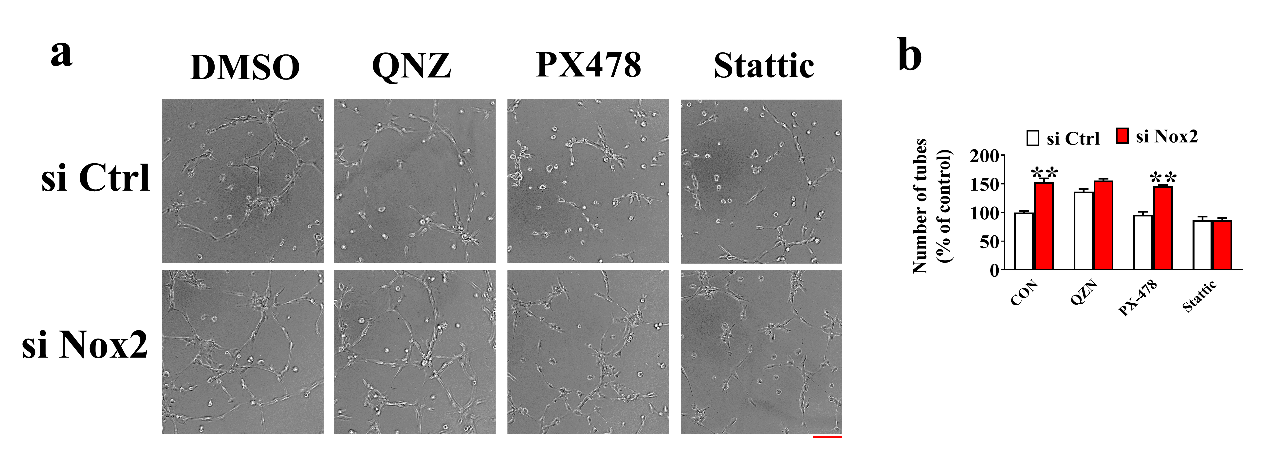
 Supplemental Fig. S6. Inhibition of STAT3 phosphorylation reduces the tube formation of PVECs. a. The representative images of tube formation of PVECs from the siRNA transfected cells treated with or without inhibitors for 6 h; scale bars, 100 μm. QNZ, PX478, and Stattic represent NF-κB, HIF-1α, and STAT3 inhibitor, respectively. b. Summarized data of tube formation (n=5).


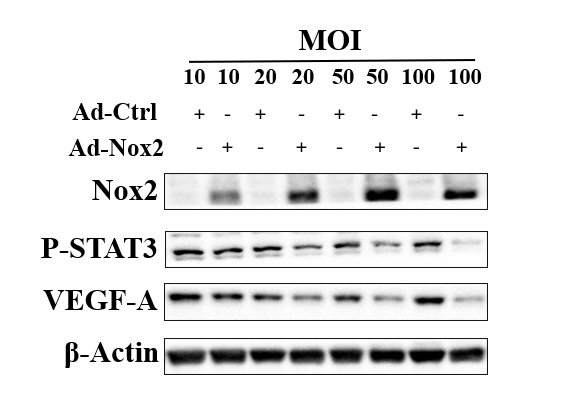


Supplemental Fig. S7. The protein levels in cells after transfection with Ad-Ctrl or Ad-Nox2 adenovirus. Cells were infected with adenovirus of different multiplicity of infection (MOI). After infection for 6 h, the medium was replaced with RIPM1640 containing 10% FBS and then incubated for 48 h. Finally, Western blotting was used to assess the protein expression levels.


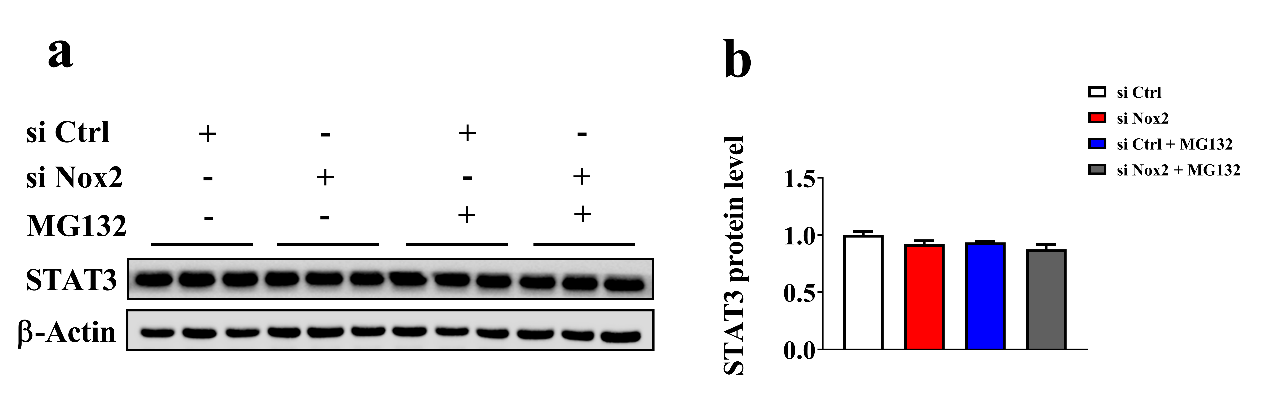
Supplemental Fig. S8. Immunoblots of STAT3 expression in PVECs. a. PVECs with Nox2 knockdown were treated with 20 µM MG132 for 4 h, then Western blotting was used to assess the protein expression levels of STAT3, with β-Actin used as the loading control; b. Summarized data of STAT3 protein levels (n=3).
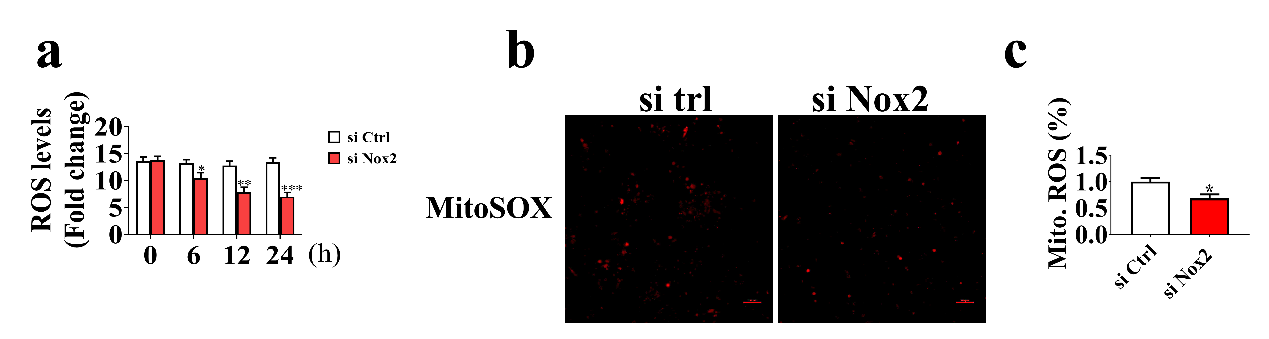


Supplemental Fig. S9. Knockdown of Nox2 reduces the formation of ROS and mitochondrial ROS. a. ROS level in PVECs transfected with si Ctrl or si Nox2 (n=5). b. Representative fluorescence images of MitoSOX stained cells; scale bar: 100 μm. c. Summarized data of the mitochondrial ROS level (n=5).
